# Expression of transforming growth factor beta mRNA isoforms in human breast cancer.

**DOI:** 10.1038/bjc.1994.197

**Published:** 1994-06

**Authors:** J. MacCallum, J. M. Bartlett, A. M. Thompson, J. C. Keen, J. M. Dixon, W. R. Miller

**Affiliations:** University Department of Surgery, Edinburgh Royal Infirmary, UK.

## Abstract

**Images:**


Br. J. Cancer (1994), 69, 1006–1009
© Macmillan Press Ltd., 1994

### Expression of transforming growth factor beta mRNA isoforms in human breast cancer

J. MacCallum[1,2], J.M.S. Bartlett[3], A.M. Thompson[1], J.C. Keen[3], J.M. Dixon[2] & W.R. Miller[2]

[1]*University Department of Surgery, Edinburgh Royal Infirmary, Lauriston Place, Edinburgh EH8 9AG, UK;* [2]*University Department of Clinical Oncology, Western General Hospital, Edinburgh EH4 2XU, UK;* [3]*ICRF Medical Oncology Unit, Western General Hospital, Edinburgh EH4 2XU, UK*

**Summary** Using an RNAse protection assay, expression of messenger RNA for isoforms of TGF-$\beta$ was determined in a series of breast cancers. Of 50 tumours, 45 (90%) expressed TGF-$\beta_1$ mRNA, 39 (78%) expressed TGF-$\beta_2$, and 47 (94%) expressed TGF-$\beta_3$. Patterns of expression varied between different tumours: 37 (74%) cancers expressed all three TGF-$\beta$ isoforms, ten (20%) expressed only two isoforms and two expressed TGF-$\beta_1$ alone. One sample showed no evidence of TGF-$\beta$ mRNA expression. Although most breast cancers expressed mRNA for at least one isoform of TGF-$\beta$, there were differences in patterns of mRNA expression between individual tumours. The relatively small number of tumours examined precluded detailed analysis between expression and other clinical parameters, but a significant association was identified between one aspect of isoform expression and lymph node status, in that the majority of tumours expressing all three isoforms were associated with lymph node involvement, whereas tumours without one or more isoform were usually lymph node negative ($P = 0.025$ by Fisher's exact test).

The transforming growth factor betas (TGF-$\beta$s) are a family of polypeptides which have important regulatory roles in a diverse series of processes including angiogenesis, embryogenesis, inflammation and immunosuppression (Roberts *et al.*, 1987, 1990; Roberts & Sporn, 1989) within both normal and transformed cells and tissues (Massague, 1987). There is evidence of altered expression of TGF-$\beta$s in cancers (Travers *et al.*, 1988; Barrett-Lee *et al.*, 1990), and certain tumours may both synthesise and respond to TGF-$\beta$s (Hsuan, 1989). It has thus been suggested that the growth of these tumours may be influenced by autocrine secretion of TGF-$\beta$ (Roberts *et al.*, 1988), and that dysregulation of TGF-$\beta$ secretion may occur in certain cancers (Lippman *et al.*, 1987; Knabbe *et al.*, 1991). It may also be relevant that the anti-oestrogen tamoxifen, which inhibits the growth of hormone-dependent tumours, can modify TGF-$\beta$ expression in breast tissues (Salomon *et al.*, 1989; Sporn *et al.*, 1990; Mizukami *et al.*, 1991; McCune *et al.*, 1992).

While most studies in TGF-$\beta$ in breast cancer have been restricted to analysis of TGF-$\beta_1$, it is evident from work with cell lines that breast cancers may express other isoforms (Arrick *et al.*, 1990) that have biological activity (Zugmaier *et al.*, 1989; Colletta *et al.*, 1990), and that patterns of expression vary between cell lines (Arrick *et al.*, 1990). The aim of this study was to examine the expression of mRNA for the three different isoforms of TGF-$\beta$ in a series of human breast cancers.

### Materials and methods

### *Tissues*

Samples were obtained from 50 patients (age range 41–91 years) presenting with histologically proven primary breast cancer to the Edinburgh Breast Unit. Fresh tumour tissue was obtained from wedge biopsy, wide excision or mastectomy specimens, snap frozen within 30 min of removal and stored in liquid nitrogen for later RNA extraction.

The oestrogen receptor status of the tumours was determined as described previously by Hawkins *et al.* (1975, 1987) with a value of $>20$ fmol per mg of protein cytosol being regarded as positive (Anderson *et al.*, 1989). Tumour stage was determined on clinical examination, and tumour type and lymph node status were obtained from the pathology reports. Tumour stage ranged from T1 to T4 (1, 28, 6 and 13 respectively) and tumours were histologically classified as 37 invasive ductal carcinomas of no special type, six invasive lobular carcinomas and seven of various special types. Lymph node involvement was determined by histological examination of axillary node sample or clearance specimens. Of the patients, 17 had been treated with tamoxifen and one with goserelin, and the remainder were untreated.

### *RNA extraction*

Total RNA was extracted using a modification of the method of Auffrey and Rougeon (1980). Frozen tumour tissue (minimum weight 0.4 g) was dismembranated in liquid nitrogen, suspended in 3 M lithium chloride–6 M urea (6 ml), sonicated and nucleic acids precipitated overnight at 4°C. Total RNA was recovered by centrifugation (15,000 *g*), and resuspended in 10 mM Tris/0.5% SDS (6 ml) containing proteinase K (50 $\mu$g ml$^{-1}$) and incubated at 37°C for 20 min. Protein was removed using 100% phenol (pre-equilibrated with 0.1 M Tris, pH 7.4) followed by phenol–chloroform–isoamyl alcohol (25:24:1 v/v/v) and 100% chloroform by centrifugation, with the aqueous phase recovered on each occasion. The RNA was precipitated overnight at −80°C in lithium chloride (300 $\mu$l, 8 M) and absolute alcohol (2.5 volumes) then recovered by centrifugation (4,000 *g*, 4°C for 45 min) and resuspended in diethyl pyrocarbonate-treated water. RNA content and purity were assessed by spectrophotometry at 260 nm and 280 nm. RNA aliquots (20 $\mu$g) were stored at −80°C or in liquid nitrogen until assayed.

### *Synthesis of riboprobes*

Specific probes were prepared as described by Bartlett *et al.* (1992) using a Gemini II system (Promega, UK), and full-length transcripts were isolated by polyacrylamide electrophoresis. The probe bands were eluted from the gel and resuspended in hybridisation buffer for use in the RNAse protection assay.

### *RNAse protection assay*

This was carried out as described in Bartlett *et al.* (1992). Owing to the similarity in sizes of TGF-$\beta_1$ and TGF-$\beta_2$, and of TGF-$\beta_3$ and actin, these pairs of probes were not added to the same sample aliquot for hybridisation. For those samples to which TGF-$\beta_3$ was added, another aliquot containing the

Correspondence: J. MacCallum
Received 8 September 1993; and in revised form 24 January 1994

full transcript for actin was analysed in parallel on a separate gel, thus ensuring the integrity of the RNA extract. Sample–probe hybrids remaining after digestion with RNAse were denatured and separated by gel electrophoresis.

*Sample scoring and statistics*

Sample RNA extracts were considered positive for each TGF-$\beta$ isoform when a band of the appropriate length (full-length transcript) was observed in the presence of a positive transcript for control actin. Absence of such a band was considered a negative result. Expression of each TGF-$\beta$ mRNA isoform and expression patterns were related to clinical, biochemical and histological parameters and associations were examined using Fisher's exact test.

### Results

Representative autoradiographs from RNAse protection assays for each of the isoforms of TGF-$\beta$ are shown in Figure 1. In the autoradiographs probe standards are visible as bands migrating slightly above the level of the corresponding sample bands. This is due to the continued presence of polylinked ends on the probe standards. tRNA controls were run on each gel and are negative for all probes. Sample RNA extracts were examined for bands corresponding to the TGF-$\beta$ probe added. Of the 50 tumours studied, 45 showed expression of mRNA for TGF-$\beta_1$, 39 expressed mRNA for TGF-$\beta_2$, and 47 expressed mRNA for TGF-$\beta_3$.

Despite high rates of expression for all three isoforms, different patterns of expression were evident in individual tumours (Table I). The majority of tumours (37) expressed all isoforms of TGF-$\beta$, ten expressed only two isoforms (eight expressing TGF-$\beta_1$ AND TGF-$\beta_3$; two expressing TGF-$\beta_2$ and TGF-$\beta_3$) and two expressed TGF-$\beta_1$ alone. One tumour did not appear to express mRNA for any isoform, although a positive signal was obtained for control actin.

Oestrogen receptor (ER) status was measured in 48 of the 50 tumours, of which 30 were ER positive and 18 ER negative. Correlations between expression of individual mRNA isoforms and oestrogen receptor status are shown in Table II. None of the relationships was statistically significant, although tumours not expressing either TGF-$\beta_2$ or -$\beta_3$ mRNA tended to be ER positive.

There were 21 histological lymph node (LN)-positive and 20 lymph node-negative tumours; in another nine tumours the axillary node status was not determined histologically. The relationships between lymph node status and TGF-$\beta$ isoform expression are shown in Table III. These again were non-significant, although showing a trend for tumours without expression of individual TGF-$\beta$ isoforms to be LN negative. The data were also analysed as a comparison of tumours which express mRNA for all three TGF-$\beta$ isoforms *vs* those that do not (Table IV). This identified a significant relationship, with the majority (57%) of tumours expressing all three isoforms being associated with lymph node involvement, whereas most tumours (82%) from patients whose tumours were without one or more isoforms were more likely to be LN negative.

There were no correlations evident between TGF-$\beta$ expression patterns and treatment status.

### Discussion

The effects of TGF-$\beta$s on tumour growth are controversial, in that, while they may directly inhibit proliferation of epithelial cancer cells (Lippman *et al.*, 1987; Knabbe *et al.*, 1991), TGF-$\beta$s may also promote tumour growth by their angiogenic and immunosuppressive properties (Roberts *et al.*, 1988; Salomon *et al.*, 1989) and effects on the extracellular matrix.

In the breast, several studies which have compared levels of TGF-$\beta$ mRNA in normal, benign and malignant tissue have suggested that values may be higher in malignant than non-malignant tissue (Travers *et al.*, 1988; Barrett-Lee *et al.*, 1990). However, these investigations did not distinguish between isoforms of TGF-$\beta$, although all three human TGF-$\beta$ mRNA isoforms have been detected in breast cancer cell

**Figure 1** TGF-$\beta$ mRNA expression in human breast cancer. Representative autoradiographs showing expression of the three human TGF-$\beta$ mRNA isoforms as determined by RNAse protection assay.

**Table I** Summary of TGF-$\beta$ mRNA expression patterns

| | | |
|---|---|---|
| *Three isoforms* | | |
| TGF-$\beta_1$ + $\beta_2$ + $\beta_3$ | 37 | (74%) |
| *Two isoforms* | | |
| TGF-$\beta_1$ + $\beta_3$ | 8 | (16%) |
| TGF-$\beta_2$ + $\beta_3$ | 2 | (4%) |
| *One isoform* | | |
| TGF-$\beta_1$ | 2 | (4%) |
| *No isoforms* | | |
| $\gamma$-Actin | 1 | (2%) |
| Total samples | 50 | (100%) |

**Table II** Expression of TGF-$\beta$ mRNA in oestrogen receptor-positive and -negative breast cancers

| | | Oestrogen receptor + | Oestrogen receptor − | |
|---|---|---|---|---|
| TGF-$\beta_1$ | + | 28 | 17 | $P = 1.66$ |
| | − | 2 | 1 | |
| TGF-$\beta_2$ | + | 21 | 16 | $P = 0.63$ |
| | − | 9 | 2 | |
| TGF-$\beta_3$ | + | 27 | 18 | $P = 0.65$ |
| | − | 3 | 0 | |

**Table III** Expression of TGF-$\beta$ mRNA in tumours from patients with and without lymph node involvement

| | | Lymph node involvement + | Lymph node involvement − | |
|---|---|---|---|---|
| TGF-$\beta_1$ | + | 21 | 17 | $P = 0.28$ |
| | − | 0 | 3 | |
| TGF-$\beta_2$ | + | 19 | 13 | $P = 0.25$ |
| | − | 2 | 7 | |
| TGF-$\beta_3$ | + | 21 | 18 | $P = 0.46$ |
| | − | 0 | 2 | |

**Table IV** Lymph node involvement in patients with tumours expressing three or fewer than three TGF-$\beta$ mRNA isoforms

| | Lymph node involvement − | Lymph node involvement + | |
|---|---|---|---|
| Expression of three TGF-$\beta$ mRNAs | 11 | 19 | $P = 0.025$ |
| Expression of two or fewer TGF-$\beta$ mRNAs | 9 | 2 | |

lines (Arrick *et al.*, 1990; Jeng & Jordan, 1991; Arteaga & Coffey, 1992). Expression of TGF-$\beta$ in breast cancer at the level of the mRNA (Thompson *et al.*, 1990, 1991; Delvenne *et al.*, 1992; Dalal *et al.*, 1993) has been reported, but to our knowledge the present investigation is the first in which all three human TGF-$\beta$ mRNA isoforms have been examined systematically in a series of human breast cancers.

It is of interest that high rates of expression for all isoforms of mRNA were observed, and that few tumours failed to express TGF-$\beta_1$ and TGF-$\beta_3$ mRNA. A further novel observation is that the pattern of isoform expression differed between individual tumours, and it was possible to identify tumours expressing one, two or three isoforms. Nevertheless, there appeared to be some hierachical order within the expression patterns. While the majority of tumours expressed all three isoforms of TGF-$\beta$, amongst those displaying only two isoforms, TGF-$\beta_3$ mRNA was always present and the combination of TGF-$\beta_1$ and TGF-$\beta_2$ was not seen. In addition, in the two tumours expressing only one isoform, this was TGF-$\beta_1$. The biological significance of this differential expression remains to be determined.

More detailed analysis of the present results uncovered a statistically significant correlation which indicated that the majority of tumours expressing all three isoforms of TGF-$\beta$ were derived from patients who had lymph node metastases, whereas tumours without one or more isoform tended to be LN negative. The underlying nature of this association is undefined. It may be that more aggressive tumours have a tendency coincidentally to express more forms and types of growth factor, or increased TGF-$\beta$ expression may be associated with the metastatic phenotype. In this respect it ought to be emphasised that these findings are not based on consistent non-expression of a particular isoform. While isoforms of TGF-$\beta$ may have overlapping biological activity, receptor studies suggest the existence of cell types that preferentially or specifically bind to one of the TGF-$\beta$ isoforms (Roberts *et al.*, 1988), thus cell responses may be unique to one of the receptor subtypes (Colletta, 1990).

The present results are not compatible with those previously reported by Barrett-Lee *et al.* (1990), which suggest that TGF-$\beta_1$ mRNA expression is associated with absence of lymph node metastases. In the present study, the few tumours that did not apparently express mRNA for TGF-$\beta$ were usually associated with the absence of lymph node involvement. However, it may be pertinent that, whereas Barrett-Lee *et al.* (1990) attempted quantification of mRNA, our study is based simply upon a qualitative assessment. Equally, there is considerable evidence to suggest that the quantitative levels of mRNA for TGF-$\beta$s do not closely correlate with protein expression (Kehrl *et al.*, 1986; Assoian *et al.*, 1987; Ikeda *et al.*, 1987; Kim *et al.*, 1992) and that, in these circumstances, qualitative association of mRNA expression may be more informative. Our observations would, however, support those reports which suggest a role for TGF-$\beta$ in mammary cancer metastasis (Welch *et al.*, 1990; Gorsch *et al.*, 1993) in which immunohistochemical staining for TGF-$\beta_1$ (but not TGF-$\beta_2$ and $\beta_3$) was associated with rate of disease progression (Gorsch *et al.*, 1992). Similarly, Walker and Dearing (1992) observed that staining for TGF-$\beta_1$ protein in primary breast cancers was associated with metastatic spread to axillary lymph nodes. Further evidence of a role for TGF-$\beta$ in tumour spread comes in a recent immunocytochemical study in which TGF-$\beta_1$ was localised at the growing edges of primary cancers (Dalal *et al.*, 1993). However, further studies, examining a larger number of samples, analysing both levels of expression and determining the biological activity of different isoforms of TGF-$\beta$ are required before definitive conclusions can be reached regarding the influence of TGF-$\beta$ expression on tumour progression.

Although the present study has shown that different TGF-$\beta$ mRNA isoforms are expressed by human breast cancers, it is not possible, using the methodology described, to identify the cell types responsible for their production. Studies in lactating and non-lactating bovine mammary tissue suggest that there is a different spatial distribution for each of the three TGF-$\beta$ isoforms (Maier *et al.*, 1991). Immunohistochemistry has been used to identify the site of TGF-$\beta$ protein, but the results to date are confusing, with McCune *et al.* (1992) suggesting that in a variety of benign and malignant breast tissues TGF-$\beta$ tends to be associated intracellularly within epithelial cells rather than stroma, and Butta *et al.* (1992) indicating TGF-$\beta$ is predominantly seen in the stroma. Given the potential role of TGF-$\beta$ in normal and malignant breast tissue and the changes in TGF-$\beta$ which have been reported after treatment with tamoxifen, it is important to resolve such anomalies. Our current studies employing immunohistochemistry and *in situ* hybridisation aim to clarify these issues.

The authors would like to thank the pathologists of the Breast Unit for their help in collecting tumours and histopathological assessment, and Professor D.C. Carter for his interest in this study. We are also grateful to the Scottish Hospital Endowments Research Trust for their financial support (Grant: SHERT 1092).

### References

ANDERSON, E.D.C., FORREST, A.P.M., LEVACK, P.A., CHETTY, U. & HAWKINS, R.A. (1989). Response to endocrine manipulation and oestrogen receptor concentration in larger operable primary breast cancer. *Br. J. Cancer*, **60**, 223–226.

ARRICK, B.A., KORE, M. & DERYNCK, R. (1990). Differential regulation of expression of three transforming growth factor $\beta$ species in human breast cancer cell lines by estradiol. *Cancer Res.*, **50**, 299–303.

ARTEAGA, C.L. & COFFEY, R.J. (1992). Transforming growth factor-β isoforms in mammary neoplasia: more questions than answers. *Hum. Pathol.*, **23**, 1-3.

ASSOIAN, R.K., FLEURDELYS, B.E., STEVENSON, H.C., MILLER, P.J., MADTES, D.K., RAINES, E.W., ROSS, R. & SPORN, M.B. (1987). Expression and secretion of type β transforming growth factor by activated human macrophages. *Proc. Natl Acad. Sci. USA*, **84**, 6020-6024.

AUFFRAY, C. & ROUGEON, F. (1980). Purification of mouse immunoglobulin heavy chain messenger RNA from total myeloma tumour RNA. *Eur. J. Biochem.*, **107**, 303.

BARRETT-LEE, P., TRAVERS, M., LUQMANI, Y. & COOMBES, R.C. (1990). Transcripts for transforming growth factors in human breast cancer: clinical correlates. *Br. J. Cancer*, **61**, 612-617.

BARTLETT, J.M.S., RABIASZ, G.J., SCOTT, W.N., LANGDON, S.P., SMYTH, J.F. & MILLER, W.R. (1992). Transforming growth factor-β mRNA expression and growth control of human ovarian carcinoma cells. *Br. J. Cancer*, **65**, 655-660.

BUTTA, A., MACLENNAN, K., FLANDERS, K.C., SACKS, N.P.M., SMITH, I., MCKINNA, A., DOWSETT, M., WAKEFIELD, L.M., SPORN, M.B., BAUM, M. & COLLETTA, A.A. (1992). Induction of transforming growth factor β1 in human breast cancer *in vivo* following tamoxifen treatment. *Cancer Res.*, **52**, 4261-4264.

COLLETTA, A.A. (1990). The transforming growth factors beta - their potential in the treatment and chemoprevention of cancer. *Cancer Topics*, **8**, 18-19.

DALAL, B.I., KEOWN, P.A. & GREENBERG, A.H. (1993). Immunocytochemical localization of secreted transforming growth factor-β to the advancing edges of primary tumours and to lymph node metastases of human mammary carcinoma. *Am. J. Pathol.*, **143**, 381-389.

DELVENNE, C.G., WINKLER-GOL, R.A., PICCART, M.J., HUSTIN, J., MICHAUX, D., LECLERQ, G., NOGARET, J.M. & AUTIER, Ph. (1992). Expression of c-erb-B2, TGF-$\beta_1$ and pS2 genes in primary human breast cancers. *Eur. J. Cancer*, **28**, 700-705.

GORSCH, S.M., MEMOLI, V.A., STUKEL, T.A., GOLD, L.I. & ARRICK, B.A. (1992). Immunohistochemical staining for transforming growth factor β1 associates with disease progression in human breast cancer. *Cancer Res.*, **52**, 6949-6952.

GORSCH, S., MEMOLI, V., STUKEL, T., GOLD, L. & ARRICK, B. (1993). Immunohistochemical staining for transforming growth factor β1 associates with disease progression in breast cancer. *Proc. Am. Assoc. Breast Cancer*, **34**, 190.

HAWKINS, R.A., HILL, A. & FREEDMAN, B. (1975). A simple method for the determination of oestrogen receptor concentrations in breast tumours and other tissues. *Clin. Chim. Acta*, **64**, 203-210.

HAWKINS, R.A., SANGSTER, K., TESDALE, A.L., FERGUSON, W.A., KRAJEWSKI, A., LEVACK, P.A. & FORREST, P. (1987). Experience with new assays for oestrogen receptors using monoclonal antibodies. *Biochem. Soc. Trans.*, **15**, 949-950.

HSUAN, J.J. (1989). Transforming growth factors beta. *Br. Med. Bull.*, **45**, 425-437.

IKEDA, T., LIOUBIN, M.N. & MARQUARDT, H. (1987). Human transforming growth factor type β2: production by a prostatic adenocarcinoma cell line, purification, and initial characterisation. *Biochemistry*, **26**, 2406-2410.

JENG, M.-H. & JORDAN, V.C. (1991). Growth stimulation and differential regulation of transforming growth factor-$\beta_1$ (TGF$\beta_1$), TGF$\beta_2$ and TGF$\beta_3$ messenger RNA levels by norethindrone in MCF-7 human breast cancer cells. *Mol. Endocrinol.*, **5**, 1120-1128.

KEHRL, J.H., WAKEFIELD, L.M., ROBERTS, A.B, JAKOWLEW, S., ALVAREZ-MON, M., DERYNCK, R., SPORN, M.B. & FAUCI, A.S. (1986). Production of transforming growth factor β by human T lymphocytes and its potential role in the regulation of T cell growth. *J. Exp. Med.*, **163**, 1037-1050.

KIM, S.-J., PARK, K., KOELLER, D., KIM, K.Y., WAKEFIELD, L.M., SPORN, M.B. & ROBERTS, A.B. (1992). Post-transcriptional regulation of the human transforming growth factor-β1 gene. *J. Biol. Chem.*, **267**, 13702-13707.

KNABBE, C., ZUGMAIER, G., SCHMAHL, M., DIETEL, M., LIPPMAN, M.E. & DICKSON, R.B. (1991). Induction of transforming growth factor β by the antioestrogens droloxifene, tamoxifen and toremifene in MCF-7 cells. *Am. J. Clin. Oncol.*, **14** (Suppl. 2), S15-S20.

LIPPMAN, M.E., DICKSON, R.B., GELMANN, E.P., ROSEN, N., KNABBE, C., BATES, S., BRONZERT, D., HUFF, K. & KASID, A. (1987). Growth regulation of human breast carcinoma occurs through regulated growth factor secretion. *J. Cell. Biochem.*, **35**, 1-16.

MCCUNE, B.K., MULLIN, B.R., FLANDERS, K.C., JAFFURS, W.J., MULLEN, L.T. & SPORN, M.B. (1992). Localization of transforming growth factor-β isotypes in lesions of the human breast. *Hum. Pathol.*, **23**, 13-20.

MAIER, R., SCHMID, P., COX, D., BILBE, G. & MCMASTER, G.K. (1991). Localisation of transforming growth factor-$\beta_1$, -$\beta_2$ and -$\beta_3$ gene expression in bovine mammary gland. *Mol. Cell. Endocrinol.*, **82**, 191-198.

MASSAGUÉ, J. (1987). The TGF-β family of growth and differentiation factors. *Cell*, **49**, 437-438.

MIZUKAMI, Y., TAJIRI, K., NONOMURA, A., NOGUCHI, M., TANIYA, T., KOYASAKI, N., NAKAMURA, S. & MATSUBARA, F. (1991). Effects of tamoxifen, medroxyprogesterone acetate and estradiol on tumour growth and oncogene expression in MCF-7 breast cancer cell line transplanted into nude mice. *Anticancer Res.*, **11**, 1333-1338.

ROBERTS, A.B. & SPORN, M.B. (1989). Regulation of endothelial cell growth architecture and matrix synthesis by TGF-β. *Am. Rev. Respir. Dis.*, **140**, 1126-1128.

ROBERTS, A.B., FLANDERS, K.C., KONDAIAH, P., THOMPSON, N.L., VAN OBBERGHEN-SCHILLING, E., WAKEFIELD, L., ROSSI, P., DE CROMBRUGGHE, B., HEINE, U. & SPORN, M.B. (1987). Transforming growth factor β: biochemistry and roles in embryogenesis, tissue repair and remodelling and carcinogenesis. *Recent Prog. Horm. Res.*, **44**, 157-193.

ROBERTS, A.B., THOMPSON, N.L., HEINE, U., FLANDERS, C. & SPORN, M.B. (1988). Transforming growth factor-β: possible roles in carcinogenesis. *Br. J. Cancer*, **57**, 594-600.

ROBERTS, A.B., FLANDERS, K.C., HEINE, U.I., JACKOWLEW, S., KONDAIAH, P., KIM, S.-J. & SPORN, M.B. (1990). Transforming growth factor-β: multifunctional regulator of differentiation and development. *Phil. Trans. R. Soc. Lond.*, **B327**, 145-154.

SALOMON, D.S., CIARDIELLO, F., VALVERIUS, E., SAEKI, T. & KIM, N. (1989). Transforming growth factors in human breast cancer. *Biomed. Pharmacother.*, **43**, 661-667.

SPORN, M.B., ROBERTS, A.B., WAKEFIELD, L.M., GLICK, A.B. & DANIELPOUR, D. (1990). Transforming growth factor-beta and suppression of carcinogenesis. In *Genetic Basis for Carcinogenesis: Tumour Suppression Genes and Oncogenes*. Knudson Jr, A.G., Stanbridge, E.J., Sugimura, T., Terada, M. & Watanabe, S. (eds) pp. 259-266. Japan Science Society Press: Tokyo, and Taylor & Francis: London.

THOMPSON, A.M., STEEL, C.M., FOSTER, M.E., KERR, D., PATERSON, D., DEANE, D., HAWKINS, R.A., CARTER, D.C. & EVANS, H.J. (1990). Gene expression in oestrogen-dependent human breast cancer xenograft tumours. *Br. J. Cancer*, **62**, 78-84.

THOMPSON, A.M., KERR, D.J. & STEEL, C.M. (1991). Transforming growth factor β1 is implicated in the failure of tamoxifen therapy in breast cancer. *Br. J. Cancer*, **63**, 609-614.

TRAVERS, M.Y.T., BARRETT-LEE, P.J., BERGER, U., LUQMANI, Y.A., GAZET, J.-C., POWLES, T.J. & COOMBES, R.C. (1988). Growth factor expression in normal, benign, and malignant breast tissue. *Br. Med. J.*, **296**, 1621-1624.

WALKER, R.A. & DEARING, S.J. (1992). Transforming growth factor $\text{beta}_1$ in ductal carcinoma *in situ* and invasive carcinomas of the breast. *Eur. J. Cancer*, **28**, 641-644.

WELCH, D.R., FABRA, A. & NAKAJIMA, M. (1990). Transforming growth factor β stimulates mammary adenocarcinoma cell invasion and metastatic potential. *Proc. Natl Acad. Sci. USA*, **87**, 7678-7682.

ZUGMAIER, G., ENNIS, B.W., DESCHAUER, B., KATZ, D., KNABBE, C., WILDING, G., DALY, P., LIPPMAN, M.E. & DICKSON, R.B. (1989). Transforming growth factors type $\beta_1$ and $\beta_2$ are equipotent growth inhibitors of human breast cancer cell lines. *J. Cell Physiol.*, **141**, 353-361.


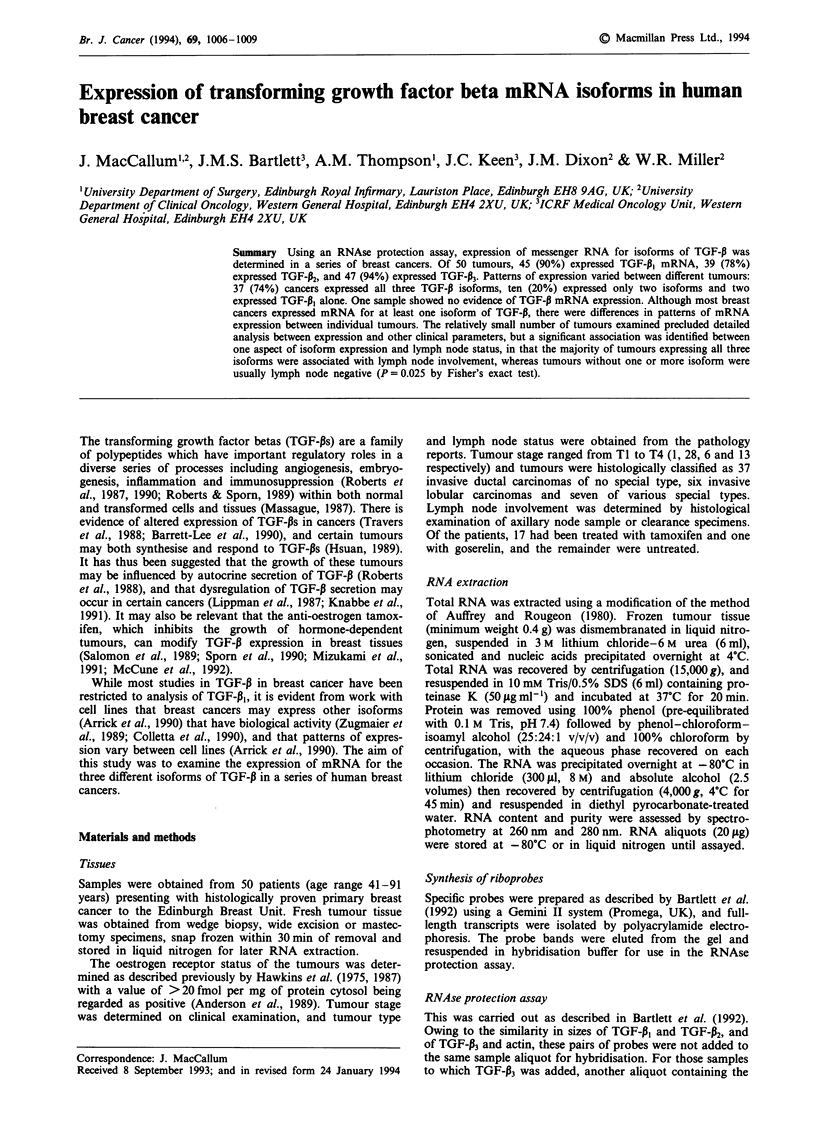

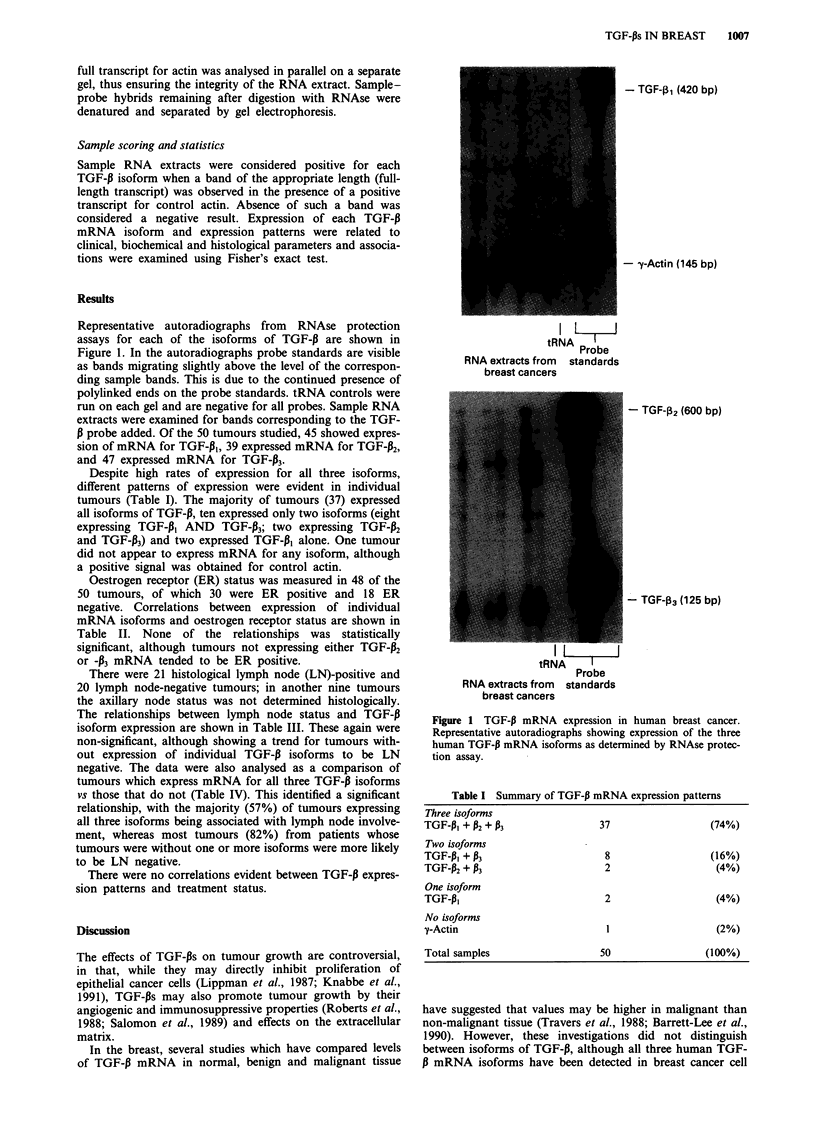

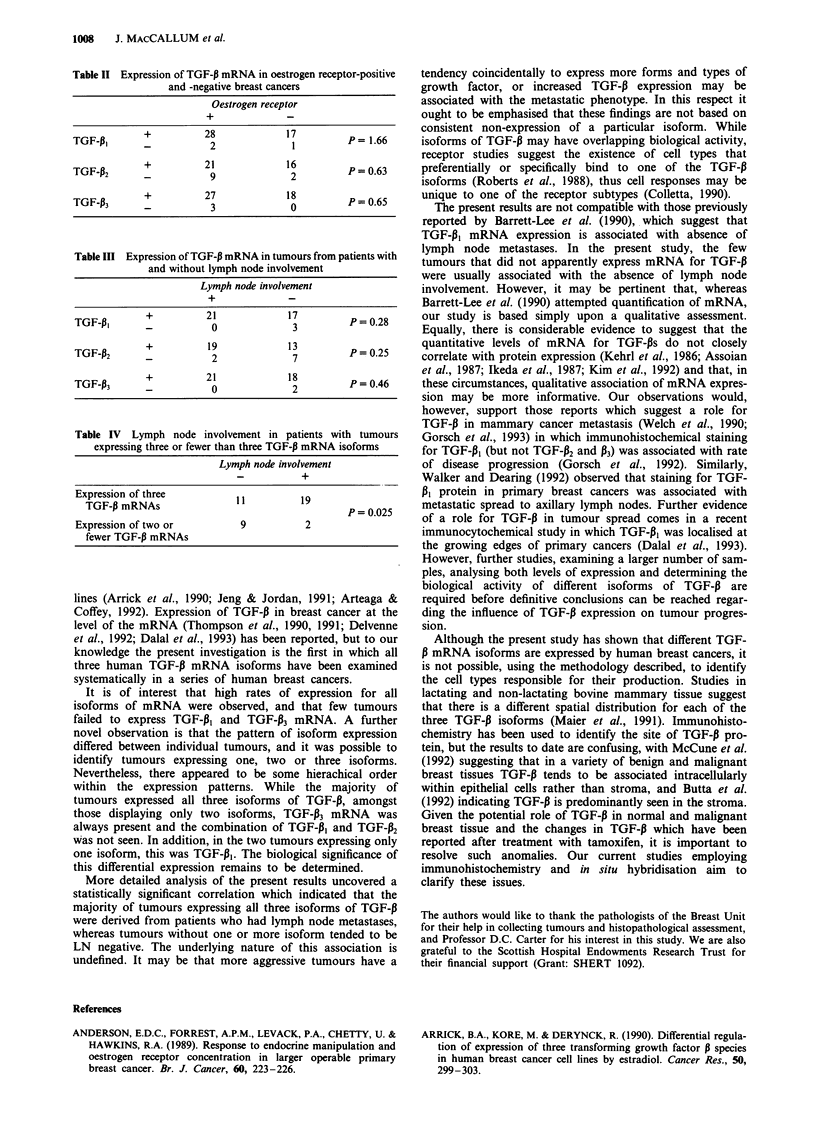

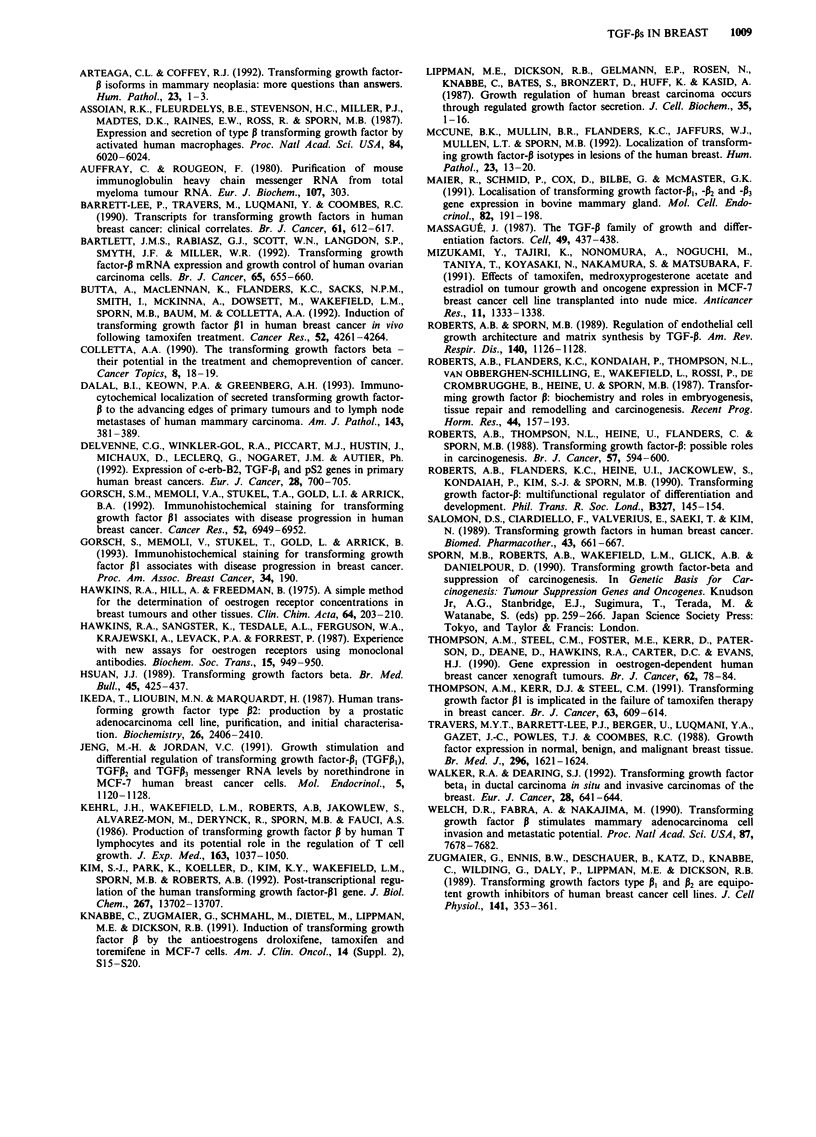


## References

[OCR_00411] Anderson E. D., Forrest A. P., Levack P. A., Chetty U., Hawkins R. A. (1989). Response to endocrine manipulation and oestrogen receptor concentration in large operable primary breast cancer.. Br J Cancer.

[OCR_00417] Arrick B. A., Korc M., Derynck R. (1990). Differential regulation of expression of three transforming growth factor beta species in human breast cancer cell lines by estradiol.. Cancer Res.

[OCR_00425] Arteaga C. L., Coffey R. J. (1992). Transforming growth factor-beta isoforms in mammary neoplasia: more questions than answers.. Hum Pathol.

[OCR_00430] Assoian R. K., Fleurdelys B. E., Stevenson H. C., Miller P. J., Madtes D. K., Raines E. W., Ross R., Sporn M. B. (1987). Expression and secretion of type beta transforming growth factor by activated human macrophages.. Proc Natl Acad Sci U S A.

[OCR_00437] Auffray C., Rougeon F. (1980). Purification of mouse immunoglobulin heavy-chain messenger RNAs from total myeloma tumor RNA.. Eur J Biochem.

[OCR_00442] Barrett-Lee P., Travers M., Luqmani Y., Coombes R. C. (1990). Transcripts for transforming growth factors in human breast cancer: clinical correlates.. Br J Cancer.

[OCR_00447] Bartlett J. M., Rabiasz G. J., Scott W. N., Langdon S. P., Smyth J. F., Miller W. R. (1992). Transforming growth factor-beta mRNA expression and growth control of human ovarian carcinoma cells.. Br J Cancer.

[OCR_00453] Butta A., MacLennan K., Flanders K. C., Sacks N. P., Smith I., McKinna A., Dowsett M., Wakefield L. M., Sporn M. B., Baum M. (1992). Induction of transforming growth factor beta 1 in human breast cancer in vivo following tamoxifen treatment.. Cancer Res.

[OCR_00465] Dalal B. I., Keown P. A., Greenberg A. H. (1993). Immunocytochemical localization of secreted transforming growth factor-beta 1 to the advancing edges of primary tumors and to lymph node metastases of human mammary carcinoma.. Am J Pathol.

[OCR_00472] Delvenne C. G., Winkler-Gol R. A., Piccart M. J., Hustin J., Michaux D., Leclercq G., Nogaret J. M., Autier P. (1992). Expression of c-erbB2, TGF-beta 1 and pS2 genes in primary human breast cancers.. Eur J Cancer.

[OCR_00478] Gorsch S. M., Memoli V. A., Stukel T. A., Gold L. I., Arrick B. A. (1992). Immunohistochemical staining for transforming growth factor beta 1 associates with disease progression in human breast cancer.. Cancer Res.

[OCR_00490] Hawkins R. A., Hill A., Freedman B. (1975). A simple method for the determination of oestrogen receptor concentrations in breast tumours and other tissues.. Clin Chim Acta.

[OCR_00500] Hsuan J. J. (1989). Transforming growth factors beta.. Br Med Bull.

[OCR_00504] Ikeda T., Lioubin M. N., Marquardt H. (1987). Human transforming growth factor type beta 2: production by a prostatic adenocarcinoma cell line, purification, and initial characterization.. Biochemistry.

[OCR_00510] Jeng M. H., Jordan V. C. (1991). Growth stimulation and differential regulation of transforming growth factor-beta 1 (TGF beta 1), TGF beta 2, and TGF beta 3 messenger RNA levels by norethindrone in MCF-7 human breast cancer cells.. Mol Endocrinol.

[OCR_00517] Kehrl J. H., Wakefield L. M., Roberts A. B., Jakowlew S., Alvarez-Mon M., Derynck R., Sporn M. B., Fauci A. S. (1986). Production of transforming growth factor beta by human T lymphocytes and its potential role in the regulation of T cell growth.. J Exp Med.

[OCR_00524] Kim S. J., Park K., Koeller D., Kim K. Y., Wakefield L. M., Sporn M. B., Roberts A. B. (1992). Post-transcriptional regulation of the human transforming growth factor-beta 1 gene.. J Biol Chem.

[OCR_00530] Knabbe C., Zugmaier G., Schmahl M., Dietel M., Lippman M. E., Dickson R. B. (1991). Induction of transforming growth factor beta by the antiestrogens droloxifene, tamoxifen, and toremifene in MCF-7 cells.. Am J Clin Oncol.

[OCR_00537] Lippman M. E., Dickson R. B., Gelmann E. P., Rosen N., Knabbe C., Bates S., Bronzert D., Huff K., Kasid A. (1987). Growth regulation of human breast carcinoma occurs through regulated growth factor secretion.. J Cell Biochem.

[OCR_00550] Maier R., Schmid P., Cox D., Bilbe G., McMaster G. K. (1991). Localization of transforming growth factor-beta 1, -beta 2 and -beta 3 gene expression in bovine mammary gland.. Mol Cell Endocrinol.

[OCR_00556] Massagué J. (1987). The TGF-beta family of growth and differentiation factors.. Cell.

[OCR_00544] McCune B. K., Mullin B. R., Flanders K. C., Jaffurs W. J., Mullen L. T., Sporn M. B. (1992). Localization of transforming growth factor-beta isotypes in lesions of the human breast.. Hum Pathol.

[OCR_00560] Mizukami Y., Tajiri K., Nonomura A., Noguchi M., Taniya T., Koyasaki N., Nakamura S., Matsubara F. (1991). Effects of tamoxifen, medroxyprogesterone acetate and estradiol on tumor growth and oncogene expression in MCF-7 breast cancer cell line transplanted into nude mice.. Anticancer Res.

[OCR_00586] Roberts A. B., Flanders K. C., Heine U. I., Jakowlew S., Kondaiah P., Kim S. J., Sporn M. B. (1990). Transforming growth factor-beta: multifunctional regulator of differentiation and development.. Philos Trans R Soc Lond B Biol Sci.

[OCR_00573] Roberts A. B., Flanders K. C., Kondaiah P., Thompson N. L., Van Obberghen-Schilling E., Wakefield L., Rossi P., de Crombrugghe B., Heine U., Sporn M. B. (1988). Transforming growth factor beta: biochemistry and roles in embryogenesis, tissue repair and remodeling, and carcinogenesis.. Recent Prog Horm Res.

[OCR_00568] Roberts A. B., Sporn M. B. (1989). Regulation of endothelial cell growth, architecture, and matrix synthesis by TGF-beta.. Am Rev Respir Dis.

[OCR_00581] Roberts A. B., Thompson N. L., Heine U., Flanders C., Sporn M. B. (1988). Transforming growth factor-beta: possible roles in carcinogenesis.. Br J Cancer.

[OCR_00592] Salomon D. S., Ciardiello F., Valverius E., Saeki T., Kim N. (1989). Transforming growth factors in human breast cancer.. Biomed Pharmacother.

[OCR_00612] Thompson A. M., Kerr D. J., Steel C. M. (1991). Transforming growth factor beta 1 is implicated in the failure of tamoxifen therapy in human breast cancer.. Br J Cancer.

[OCR_00608] Thompson A. M., Steel C. M., Foster M. E., Kerr D., Paterson D., Deane D., Hawkins R. A., Carter D. C., Evans H. J. (1990). Gene expression in oestrogen-dependent human breast cancer xenograft tumours.. Br J Cancer.

[OCR_00617] Travers M. T., Barrett-Lee P. J., Berger U., Luqmani Y. A., Gazet J. C., Powles T. J., Coombes R. C. (1988). Growth factor expression in normal, benign, and malignant breast tissue.. Br Med J (Clin Res Ed).

[OCR_00623] Walker R. A., Dearing S. J. (1992). Transforming growth factor beta 1 in ductal carcinoma in situ and invasive carcinomas of the breast.. Eur J Cancer.

[OCR_00628] Welch D. R., Fabra A., Nakajima M. (1990). Transforming growth factor beta stimulates mammary adenocarcinoma cell invasion and metastatic potential.. Proc Natl Acad Sci U S A.

[OCR_00634] Zugmaier G., Ennis B. W., Deschauer B., Katz D., Knabbe C., Wilding G., Daly P., Lippman M. E., Dickson R. B. (1989). Transforming growth factors type beta 1 and beta 2 are equipotent growth inhibitors of human breast cancer cell lines.. J Cell Physiol.

